# From the Cancer Stage of Capitalism to the Political Principle of the Common: The Social Immune Response of "Food as Commons"

**DOI:** 10.34172/ijhpm.2021.20

**Published:** 2021-03-31

**Authors:** Nick Rose

**Affiliations:** Faculty of Higher Education, William Angliss Institute, Melbourne, VIC, Australia.

**Keywords:** Food Systems, Food Politics, Global Public Health, Capitalism, Food as Commons

## Abstract

**Background:** Critical scholars agree that contemporary globalised and industrialised food systems are in profound and deepening crises; and that these systems are generative of accelerating multiple crises in the earth’s life systems. Why and how did we arrive at this point? This paper argues that, conceiving each individual human as one cell in the greater human body, we are afflicted by what John McMurtry termed ‘the cancer stage of capitalism.’ This provocative framing is adopted here in response to growing calls by climate, earth and physical scientists not to ‘mince words’ in the description and analysis of humanity’s current predicament, but rather ‘tell it like it is.’

**Methods:** Proceeding from McMurtry’s application of the seven defining medical properties of a ‘cancer invasion [of] an individual organism’ to the broader body politic and the earth’s life system, this paper draws on literature from diverse disciplines to investigate the fundamental cause of food systems crises. The paper references several empirical studies and meta-reviews that indicate the hastening decline in the integrity of human and ecological health, with a particular focus on the grain-oilseed-livestock complex and the accompanying social and ecological impacts on the southern cone countries of South America.

**Results:** The cause of food system crises is to be found in the core logic of capital accumulation, the profit imperative, and the relentless and expanding processes of commodification and financialization. The key metric of ‘economic growth’ is problematised and discussed. An embryonic ‘social immune response’ is now observable, in the diverse practices of de-commodification, proposals for de-growth and commoning that together constitute an emerging ‘food as a commons’ movement.

**Conclusion:** As currently framed, the Food as Commons proposal lacks coherence, rigour and a viable strategy to move beyond the current crisis. Its transformative potential can be strengthened through a more explicitly political grounding based on appeals to and support of anti- and post-capitalist movements and initiatives.

## Background

‘*The Great Work now [is] how we can move from a period of human devastation of the earth [and each other], to a period when we would be present to the planet [and each other] in a mutually beneficial manner.’*

**Thomas Berry**^1^

This special edition begins from the premise that ‘today’s food systems are not working for human and planetary health.’ The various contributions to the special edition validate this statement from multiple perspectives and empirical case studies. Whether we examine the factory farming of livestock, the proliferation of ultra-processed and unhealthy foods and sugary beverages, or the links between food system financialisation, large-scale land acquisitions and highly polluting agri-business, the underlying theme is clear: the contemporary global food system has generated a pandemic of non-communicable diseases and produced environmental devastation on a barely comprehensible scale.^2-5^ This sombre picture becomes bleaker still when we examine the multiple intersecting and reinforcing policy, regulatory and institutional mechanisms and dynamics by which this food system further entrenches, consolidates and expands itself, and is being expanded, through time and space. With the coronavirus disease 2019 (COVID-19) pandemic still raging globally at the time of this writing, the willingness of governments and businesses to sacrifice the health and lives of workers throughout the food system and the economy more broadly has brought into the sharpest possible relief the opposing interests of national and global public health, on the one hand; and the relentless and unrelenting drive for capital accumulation and profit, on the other – regardless of the consequences.^6,7^

This paper is organised into three parts. The first summarises the empirical evidence to substantiate the claim that the current globalised, industrialised and capitalist food system is now so destructive of human and non-human life that its transformation is a matter of urgent necessity. The second locates the cause of this accelerating destructive trajectory in the global political economy of capitalism and its core organising logics of expanding commodification and ceaseless capital accumulation,^8^ by reference *inter alia *to theoretical framework developed by John McMurtry in *The Cancer Stage of Capitalism*.^9^ McMurtry’s framework is located within the broader trajectory of ecological economics and in particular the growing scholarly and policy interest in post-growth and post-capitalist proposals. In the final section, the paper interrogates the framing of ‘food as commons’ and associated proposals and practices for the de-commodification of food systems.^10^ It is suggested that the reclamation and implementation of the ‘political principle of the common’ offers the prospect of a lasting exit from the contemporary crisis, and this paper argues for the widespread adoption and advocacy of this principle by critical public health and food system scholars and practitioners.^11^

## Key Messages

**Implications for policy makers**

- Commit to the full and universal implementation of the human right to adequate and culturally appropriate food.
- Form multi-disciplinary teams to develop coherent and feasible transition plans towards a post-growth and post-capitalist economy.
- Ensure that such plans are based on clear commitments to the principles of optimising human well-being and ecological integrity.
- Engage widely with stakeholders and community members to build understanding of the need for such plans and consensus around them.

**Implications for public**

Humanity is at a crossroads. The ways in which our societies and economies have developed over the past 150 years, and most especially the last 40 years, has brought us to the very precipice of ecological and societal collapse. The climate emergency and the coronavirus disease 2019 (COVID-19) pandemic are two of the most recent and most acute manifestations of this predicament. The contemporary globalised and industrialised food system is deeply implicated in the systemic crises we face. There is an urgent need for greater public engagement with and understanding of the systemic and interconnected nature of these issues and challenges, as well as much more discussion about alternative political economies that can help us navigate current and emerging crises.

## Part 1: The Globalising, Industrialised and Capitalist Food System and its Converging Crises

“ *The [food system is the] web of actors, processes and interactions involved in growing, processing, distributing, consuming and disposing of foods … A holistic food systems lens is concerned with how these processes interact with one another, and with the environmental, social, political and economic context. The food systems lens also brings to light reinforcing and balancing feedback loops, tensions between the different components and flows of food systems, and interactions that are cyclical, multilayered and multi-scale. It is a way of thinking about the world that seeks to identify the linear and non-linear relationships between the different components of the system” *(International Panel of Experts on Sustainable Food Systems p. 3).^12^

The holistic food systems approach emerged from the development of complex adaptive systems theory and in particular ecological systems theory according to which ‘human beings are embedded in multiple nested systems and human development is the result of the complex interactions between and within these systems over time.’^13^ With its emphasis on holism, relationality and interdependence, there is a strong affinity between food systems theory and the eco-centric cosmologies of Indigenous peoples, who ‘feel responsible for living in harmony with nature and creating a culture that reciprocally protects and nourishes life in all its forms.’^13^ Such ontologies and forms of knowing ‘differ fundamentally from the prevailing totalitarian, positivistic, rationalist, mechanistic and reductionist Western worldviews.’^13^

The contrast is particularly sharp with the globalising, industrialised and capitalist food system which, notwithstanding recent moves towards ‘zero waste,’ ‘green growth’ and ‘circular economy’ discourse, is the very embodiment of the reductionist Western worldview. There are certain common features that have characterised the capitalist food system since its commencement in the plantation economies of the Americas, rooted in the trans-Atlantic slave trade of the first centuries of European colonisation. These include time intensification (increasing the speed of production with a focus on quantity rather quality); spatial expansion and the homogenisation of natural and built environments, as well as the reduction in genetic diversity of plants and animals; a tendency towards both under- and over-consumption; the production of individual subjectivity leading to ‘possessive individualism’ and the rupturing of social and communal bonds; increasing commodification and commercialisation as well as greater levels of market dependence, resulting in the reduction of social relations to the cash nexus; and the tendency towards concentration of capital in larger units, leading to oligopolies across many sectors of the food system and the economy more broadly.^14-17^

What are the outcomes of these historically-observable phenomena over time and space? In the first instance, if the basic metric by which food systems should be assessed is achieving universal food security, the capitalist food system is failing: the lives of as many as two billion people are blighted by hunger, malnutrition and food insecurity, a number that has increased substantially in the wake of the COVID-19 pandemic.^18,19^ The highly processed and heavily marketed products of the industrialised and capitalist food system have produced an epidemic of diet-related diseases: a systematic review conducted for the 2017 *Global Burden of Disease* found that the single greatest risk factor for early death and morbidity is now diet, accounting for 11 million deaths in 2017, or one in every five.^20,21^More broadly, the human health impacts of the globalised industrial food system, from paddock to plate, incorporating not only the impacts of unhealthy diets but also occupational hazards and accidents (such as prolonged exposure of farmers and rural workers to toxic agro-chemicals), were estimated (in monetary terms) to total nearly $US13 trillion, or one-sixth of global gross domestic product in 2018.^22^

Such suffering at the individual and population level is, however, a significant commercial opportunity for the corporate players in the global healthcare market, valued at $US8.45 trillion in 2018, and, with an anticipated compounding annual growth rate of nearly 9%, expected to reach nearly $US12 trillion by 2022.^23^ In a revealing statement, a recent *Businesswire* commentary on this booming sector noted that:

“ *Going forward, faster economic growth, technological developments and the increasing prevalence of diseases due to rising busy and sedentary lifestyles will drive the growth [of the global healthcare market]. Factors that could hinder the growth of this market in the future are rising interest rates, increasing awareness of alternative therapies and natural remedies, government provisions in healthcare services, and stringent government regulations*”^23^ (emphasis added).

The implication here is that greater public spending on healthcare and better public health generally are, from the perspective of the private healthcare market, unwelcome, insofar as they inhibit increasing profit. From the standpoint of ethics and a commitment to basic human rights, including the right to the highest attainable standard of health, such reasoning can only be described as perverse. And yet it is widely accepted as the ‘common sense’ of industry and financial markets, as well as being reinforced in a directly material sense by highly effective lobbying efforts aimed at inhibiting public health measures such as a sugar tax.^24^

In terms of the ecological impacts, large-scale industrialised monocultures and the deforestation and land-use change that they entail are major drivers of anthropogenic climate change, with the food system accounting for as much as 37% of all greenhouse gas emissions, according to the Intergovernmental Panel on Climate Change.^25^ Such practices are also major drivers of the ‘unprecedented’ rapid decline in ecosystems and accelerating rate of species extinction, leading to humanity ‘eroding the very foundations of our economies, livelihoods, food security, health and quality of life worldwide;’ this being the conclusion of the most comprehensive assessment of the state of planetary ecosystems ever undertaken by the world’s leading scientists in their respective fields.^26^

Summarising these and other major datasets, 16 leading biophysical scientists, in a paper published in January 2021, stated that ‘the scale of the threats to the biosphere and all its lifeforms – including humanity – is so great that is difficult to grasp for even well-informed experts.’^27^ They added that the current political and policy responses were woefully inadequate to the extent and severity of the crisis, concluding that:

*“The gravity of the situation requires fundamental changes to global capitalism, education, and equality, which include inter alia the abolition of perpetual economic growth, properly pricing externalities, a rapid exit from fossil-fuel use, strict regulation of markets and property acquisition, reigning in corporate lobbying, and the empowerment of women.”* ^27^

Absent such thorough-going structural changes, they warned, the future in the coming decades would be ‘ghastly.’ They thus concluded with an exhortation to ‘experts in any discipline that deals with the future of the biosphere and human well-being to eschew reticence, avoid sugar-coating the overwhelming challenges and “tell it like it is.” Anything else is misleading at best, or negligent and potentially lethal for the human enterprise at worst.’^27^ This paper is written in the spirit of that academic and scientific call to arms.

## Part 2: Framing and Understanding the Crisis: Exponential Growth, or the Cancer Stage of Capitalism

This brief review of the empirical evidence indicates that the globalised, industrialised and capitalist food system is generative of extraordinary and increasing levels of human and ecological ill-health, disease and devastation. The capitalist food system cannot, of course, be understood in isolation or divorced from the broader economy: it is subject to the same imperatives and logics, above all for increasing production and consumption, and thus for capital accumulation and profit.^28,29^ The so-called ‘meatification’ of diets, or ‘the movement of meat from the periphery of human consumption patterns – where it was for the great majority of agricultural history – to the centre,’ linked to what Tony Weis calls the ‘grain-oilseed-livestock complex,’ provides a good illustration of this dynamic.^30^ In 1961 average per capita meat consumption globally was 23 kg; by 2009 that figure had risen to 42 kg per capita, which, taking into account the population increase over that period, ‘translates into a four-fold increase in world [meat production] in a mere half-century.^30^ As with all development statistics, these global figures mask large asymmetries, with the United States, Australia, Argentina and Canada averaging in excess of 100 kg per capita consumption, with Africa and South Asia at 18 kg and 7 kg respectively in 2009.^30^ Over the same period, China saw a 31-fold increase in per capita consumption, from 4 kg in 1961 to 59 kg by 2009.^30^

This growth in meat consumption has been driven by a very rapid expansion of factory farmed pigs and chicken, which are fed by grain produced on vast monocultures, in particular soybean in what has been called the ‘green deserts’ of the southern cone of South America.^31^ In Argentina alone, this ‘meatification of diets’ and the accompanying ‘ecological hoofprint’ saw the area of land dedicated to soybean cultivation increase from 38000 hectares in 1970 to 10 million hectares by 2001^32^; 16 million hectares by 2009^33^; and 18 million hectares by 2020.^34^ Globally, soybean cultivation now occupies 120 million hectares of land, or one-third of all arable cropland,^35^ with half of that production taking place in South America.^36^

The vast majority of soybean cultivated in Argentina, Paraguay and the southern states of Brazil is genetically modified to tolerate the herbicide glyphosate, manufactured by Bayer (formally Monsanto) and sold under the name RoundUp. The use of RoundUp in Argentina nearly doubled between 1997, when genetically modified soybeans were introduced, and 2011.^37^ Due to the huge acreages under cultivation, the herbicide is generally sprayed from the air. Over time, weeds have become resistant, and greater volumes of RoundUp have been applied, leading to rapidly worsening outcomes for human and ecological health. In the early 2000s, residents in areas close to soybean monocultures began reporting a range of health problems not previously observed, from miscarriages to birth defects to new forms of cancer, at rates several times higher than the national average.^37^ Despite years of protests, research and litigation by doctors and mothers’ groups who have claimed, with substantial evidence, that they are being poisoned, the coincidence of commercial interests combined with the Argentine government’s dependence on increasing soybean exports has meant that no meaningful action has been taken to regulate herbicide and pesticide use in the country. On the contrary, usage is increasing, with 525 million litres applied in 2018, an increase of over 30% since 2014.^38^

The ecological impacts are mirroring the human health impacts, with a 44% decline in beehives between 2010 and 2018^38^ and a 60% decline in honey production from 1996-2016.^39^ To bring more areas into cultivation, 2.5 million hectares of native forests were cleared in the nine years to 2009, an average of 821 hectares every day.^40^ In addition to the habitat loss and associated declines in biodiversity that such land use change produces (as noted above), annual soil loss through erosion is estimated to be as high as 30 tons per hectare, with accumulated soil nutrient loss (in nitrogen and phosphorous) exceeding 1.2 million tons by 2009.^40^

By the metrics of macroeconomics, the soybean boom in South America’s southern cone is a resounding commercial success. By the metrics of human and ecological health, it is an unmitigated and intensifying disaster.^41^ Even leaving to one side the concerns of public health and climate change scholars and advocates about the health and climate impacts of meat consumption,^42,43^ the ‘meatification of diets’ and the ‘green deserts’ of South America that they have produced provide a graphic demonstration as to why there is now an emerging scientific position that the continued policy drive for such forms of capital accumulation and profit-making, expressed as ‘economic growth’ (which, it should be noted, is embedded in the Sustainable Development Goals as Goal 8), is fundamentally incompatible with the avoidance of a ‘ghastly future’ and therefore should be abandoned as soon as possible.^27^

The assumption that economic growth is and should continue to be the principal policy goal of governments everywhere is however deeply entrenched, in the academy as in the bureaucracy, the financial press and the financial sector. Challenging this orthodoxy is no easy task. Ecological economics, a field that dates back to the beginnings of the modern environmental movement in the 1960s, proceeding through various debates and formulations in the 1970s, before arriving at a series of more clearly worked out positions in the late 1980s, has begun to do so in earnest.^44-46^ Foundational concepts and principles, such as sustainability, inter-generational equity, ecosystem services and cost-internalisation, have since become institutionalised and embedded in international agreements, most notably in the Sustainable Development Goals, adopted by the member states of the United Nations in 2015.

An important and highly influential early formulation of ecological economics was Herman Daly’s explication of the ‘steady-state’ economy through a seminal article in 1974.^47^ Daly argued that since the Earth itself was ‘approximately a steady state,’ human societies should accordingly organise their economic activity to strive for a similar level of stasis. What was not clear, however, was what form of political economy Daly believed this ‘steady state’ might be achieved under. When pressed by critics such as Richard Smith, Daly professed to be agnostic about whether his proposed steady-state economy was socialist or capitalist, however he made clear his preference for markets over central planning as a matter of ‘allocative efficiency’ of resources.^48,49^ He also suggested that economic growth was a matter of choice rather than a structural necessity, leading to critiques by Marxian scholars who argued, on the basis of Marx’s foundational analysis, that the growth imperative was central to capital accumulation, itself the heart of capitalism.^28,50-52^ This critique in turn was refuted by non-Marxian scholars who, working within an institutional economics framework, argued that the policy preference for growth was indeed an institutional choice that could be abandoned, and that a steady state capitalist economy could function quite successfully provided appropriate institutional arrangements were made.^53^

Mainstream policy proposals for a ‘Green New Deal’ have been premised on the basis that a ‘decoupling’ of material resource use, and associated pollution, from continued economic growth, is possible.^54,55^ This premise has in turn come under sustained attack in recent years, as efforts to articulate a ‘fair low-carbon transition’ have gathered pace.^56-58^ Increasingly, the very notion of ‘growth’ itself has become problematised as being at the root of the crises we face. As John Barry puts it:

“ *The green critique of orthodox economics must become a clearer critique of capitalism itself…Any planned economic contraction (in the developed world) as a response to climate change…must therefore be viewed for what this is and means: a transition away from capitalism since a non-growth/ degrowth capitalism is impossible as well as undesirable. Carbon- fuelled capitalism is destroying the planet’s life-support systems and is systematically liquidating them and calling it ‘economic growth’ … A post-growth critique must necessarily lead to a post-capitalist alternative and related political and ideological struggle.*”^59^

In the context of discursive and political struggles over endless and thus exponential economic growth, McMurty’s framing of ‘the cancer stage of capitalism’ has both explanatory and discursive power. McMurty insists that his framing is not a provocative metaphor or a rhetorical flourish. Rather, he argues that the ‘seven defining properties of a cancer invasion’ at the cellular level in an individual human being can also ‘be recognised at the level of global life-organisation [and that] this is the pathological core of our current disease condition [as a species].’^9,60,61^ The central proposition is that the exponential and metastisizing growth of capitalism, which takes place on the basis of relentless exploitation of human populations and ecosystems, mirrors in all essential respects the behaviour of cancer cells within an individual human body.^61^ An essential point for McMurtry is the inability of the host’s immune system to recognise the disease and respond effectively to it. This becomes the core of his argument that the ‘social immune system of the civil commons’ is perhaps the only mechanism available to humanity to save ourselves – and indeed the living planet – from the metastasizing political economy of contemporary capitalism.^61^

Capitalism as a form of social cancer afflicting humanity, yet which at the same time is internalised and naturalised as ‘normal’ even as its predations move us closer to ecosystem and thus social collapse, captures much that it is important about the contemporary situation. What is fails to identify is the ‘space-time compression’ of late capitalism described by David Harvey and Frederick Jameson, and the cultural and ideological consequences of the accelerated and distorted temporalities which thus characterise contemporary life.^62,63^ In the following passage, Joel Kovel succinctly explains the interplay between the dynamics of acceleration and commodification, and the cultural effects this produces:

*“The culture of advanced capital aims to turn society into addicts of commodity consumption, a condition ‘good for business’ and correspondingly bad for ecosystems. The evil is twofold, with reckless consumption leading to pollution and waste, while the addiction to commodities builds a society unable to comprehend, much less resist, the ecological crisis. Once time is bound in capitalist production, the subtle attunement to natural rhythms necessary for an ecocentric sensibility becomes thwarted. This allows the suicidal insanity of ever-expanding accumulation to appear as natural. People with mentalities warped by the casino complex are simply not going to think in terms of limits and balances, or of the mutual recognition of all beings. This helps account for the chorus of hosannas from presumably intelligent authorities at the nightmarish prospect of a doubling of economic product in the next twenty years.”* ^29^

If the accelerating biophysical and social contradictions of the capitalist food system were substantively manifesting a decade ago, the advent of the COVID-19 pandemic has brought them into sharp relief.^64^ Where-ever one turns, the pandemic and the responses to it reveal a fragile food system enmeshed in crisis. From extraordinary levels of food waste caused by supply chain disruptions, to sharply rising levels of food insecurity, to widespread injury and death resulting from exposure to the pandemic amongst highly exploited food system workers, to the origins of the virus itself linked in part to the global grain-livestock and factory farming complex, COVID-19 is a ‘wake-up call for the food system.’^65-75^ More broadly, the negligence with which governments in Europe, Britain and the United States handled the pandemic, leading to high rates of infection and death that would have been preventable had public health, rather than economic activity, been prioritised, led the British Medical Journal to accuse those in charge of ‘social murder.’^76^ It is important to note that while the burden of suffering in 2020 fell disproportionately on low-income sectors and people of colour, with as many as 500 million more people falling into poverty, the world’s billionaires experienced a bonanza year, with their collective wealth increasing by nearly $4 trillion.^77^

 Having laid bare the cause of our social and ecological malady – capitalism in its cancer stage - the question becomes: what is to be done?

## Part 3: The Political Principle of the Common

Proceeding from diagnosis to possible cure, McMurty sees cause for hope in what he calls the ‘social immune system of a consciously constructed [civil] commons of social life organisation and universal goods upon which the deeper and long-term development of humanity [has] always depended.’^9^ This ‘social immune system’ embraces the institutions and traditions that made life bearable and satisfying for growing numbers of working people emerging from the barbarity of early industrial capitalism. However, it is precisely these institutions and traditions that have been under sustained attack in recent decades.^17^

The reappearance of the commons can also be understood as a latter-day manifestation of Polanyi’s ‘double movement:’ the reassertion of ‘movements for social protection generated by the failure of the self-regulated market.’^79^ The last twenty years have seen a proliferation of literature valorising the return of the commons as a practice of creative resistance in the face of modern-day enclosures, such as privatisations and austerity budgets.^80-82^

One of the leading commons theorists and advocates, David Bollier, describes the commons as ‘a wide variety of self-organised social practices that enable communities to manage resources for collective benefit in sustainable ways…As a system of [basic needs] provisioning and governance, commons give participating members a significant degree of sovereignty and control over important elements of their everyday lives.’^83^ Bollier thus argues that ‘these more equitable, ecologically responsible and decentralised ways of meeting basic needs represent a promising new paradigm for escaping the pathologies of the Market / State order and constructing an ecologically sustainable order.’^83^ Bollier, his co-theorist Silke Helfrich and others, build on the legacy of Elinor Ostrom in conceptualising and analysizing the ‘commons’ as a set of goods or common-pool resources such as ‘the commons’ in the form of land, or a digital commons in the form of open-source software.^84,85^ Bollier and others look to these emerging diverse practices and see in them to potential to transition to a ‘market/state/commons triarchy,’ in which the market persists but the state becomes a ‘partner state’ ‘assisting not just the market sector but also the commons sector, working to ensure its health and well-being.’^84^

While Bollier argues for the transformative potential of the commons as an ongoing process that *may *at some point displace the market as the dominant mode of economic exchange and interaction, this perspective assumes the persistence of the ‘market/state order’ for an indeterminate time. Further, while Bollier acknowledges the current close affinity between the market and the state, and that therefore the state will likely be unwilling to embrace its new role as a ‘Partner State,’ there is no adequate theorisation, based on an analysis of class forces, configurations of power relations, and the dynamics of contemporary capitalism and crisis, to explain how such a transition would actually occur.

Such a theorisation, combined with a strategy is offered by Erik Olin Wright.^86,87^ Similar to the anti-totalizing Community Economies Collective forming in the wake of JK Gibson-Graham’s scholarship,^88^ Wright posits that at any particular point in time, in any given society, there is not a singular totality of ‘capitalism,’ but rather a combination of capitalism (private ownership of the means of production and market allocation of resources), statism (state ownership of the means of production and state allocation of resources) and socialism (social ownership of the means of production and socially-controlled allocation of resources).^87^ While capitalism has been the dominant form in most places, certainly over the past 40 years, socialist economic and social practices are observable in forms such as worker-owned cooperatives, community land trusts, community supported agriculture and community gardens. These are embryonic expressions of post-capitalist or proto-socialist economic and social forms which, given the inherent contradictions and tensions within capitalist social relations and a broader conjuncture characterised by the need to take large-scale coordinated action to deal with climate change, as well as manage social tensions and conflicts arising from mass unemployment due to technological change, may over time have the systemic effect of not only ‘taming’ capitalism but also ‘eroding’ it and thus bringing about its transformation.^87^ Conversely, Wright explicitly rejects the feasibility or desirability of ‘smashing’ capitalism through a revolutionary rupture, arguing by reference to history that such ruptures have resulted in authoritarian states that in practice have been the antithesis of socialism defined as ‘pervasive economic democracy.’^87^

Silvia Federici provides a longer historical perspective, noting that ‘commoning is the principle by which human beings have organised their existence for thousands of years;’ and that to ‘speak of the principle of the common’ is to speak ‘not only of small-scale experiments [but] of large-scale social formations that in the past were continent-wide.’^87^ Hence a commons-based society is neither a utopia or reducible to fringe projects, and the commons have persisted despite the many and continuing enclosures, ‘feeding the radical imagination as well as the bodies of many commoners.’^87^ Federici acknowledges that commons and practices of commoning are diverse, that many are susceptible to co-optation and many are consistent with the persistence of capitalism; indeed some, such as charities providing social services (including foodbanks) during the years of austerity budgets in the United Kingdom (2010-2015), reinforce and stabilise capitalism.^87^ What matters to Federici is the character and intentionality of the commons as anti-capitalist, as ‘a means to the creation of an egalitarian and cooperative society…no longer built on a competitive principle, but on the principle of collective solidarity [and commitments] to the creation of collective subjects [and] fostering common interests in every aspect of our lives.’^87^

Federici’s analysis resonates with the political thought and proposals developed by Dardot and Laval in their 2018 work, ‘ *On Common: Revolution in the 21st century*.’^11^ For Dardot and Laval, the common is likewise understood as a principle of political struggle, a demand for ‘real democracy’ and a major driving force behind the emerging articulation of a political vision and programme that transcends and overcomes the straitjacket logic of neoliberal ideological hegemony and its ‘policy grammar’ which appears to foreclose all alternatives and lock us forever into a capitalist realism in which ‘it is easier to imagine the end of the world than it is to imagine the end of capitalism.’^89^ Eschewing Bollier’s ‘triarchy’ of a market/state/commons coexistence, Dardot and Laval argue for a politics of the common based on an engaged citizenry that directly participates and deliberates in all decisions which impact it, and in the process not merely transforms the institutions responsible for the management of services and allocation of resources, but creates new institutions and new ways of being in the world.^11^

Dardot and Laval describe this form of politics as ‘instituent praxis’: the common, they argue, is ‘not produced but instituted.’^11^ This acknowledges the conventional understanding of Ostrom, Bollier and others of ‘the commons’ as residing in the rules – the laws – that a community establishes for the collective management and use of shared resources, but extends it much further and in a more radical direction. The essence of the commons, they argue, is not in the goods *per se *such as land or a forest or a seed bank ‘held in common,’ but rather in the process of their establishment as well as the ongoing negotiation that will surround their use and governance. Hence, Dardot and Laval distinguish the commons from the ‘rights’ tradition of property, arguing that ‘the commons are above all else matters of institution and government…the use of the commons is inseparable from the right of deciding and governing. *The practice that institutes the commons is the practice that maintains them and keeps them alive and takes full responsibility for their conflictuality through the coproduction of rules*.’^90^ To ‘institute’ in this context should not be misunderstood as ‘to institutionalise [or] render official;’ rather it is ‘ *to recreate with, or on the basis of, what already exists.*’^90^ This messy, conflictual and evolving process is what Dardot and Laval insist will ultimately bring about a revolution, not in the form of a violent uprising or insurrection, but rather through the ‘reinstitution of society’ via the transformation of politics and economy from its current state of ‘representative oligarchy’ to full participatory and deliberative democracy.^11^ Such a vision is premised on a mass politicisation of society; in effect a return of mass popular political contestation and a turn away from the post-political era of the neoliberal consumer.^91-92^

###  Food as a Commons

How do such theorisations translate to the food system, and its prospects for transformation? Some examples of food system initiatives potentially aligned with an anti- and post-capitalist trajectory, and as embodying dimensions of the commons to a greater or less extent, have been noted earlier. Silvia Federici, for example, identified ‘urban community gardens in particular as promising projects because [in some instances] they merge women’s emancipation, land redistribution and revolts against neoliberal capitalism.’^93^

In 2018, the *Routledge Handbook of Food as a Commons* was published as ‘the first comprehensive review and synthesis of knowledge and new thinking on how food and food systems can be thought, interpreted and practice around the old/new paradigms of commons and commoning.’^10^ The editors and their contributing authors agree that the re-emergence of discourses and practices of reclaiming ‘the commons’ (notably as indigenous-led resistance to egregious processes of neoliberal privatisations such as the ‘water wars’ of Cochabamba, Bolivia in 1999-2000) has occurred in reaction to the increasing commodification of food and food systems, and the negative consequences of such commodification. The editors and contributors also share an overarching premise, namely the need to transcend the treatment of food ‘as a mere commodity’^10^ because *inter alia *such reductive economistic logic is both blind and deaf to social injustice and inequality, as well as ecological devastation; and because the commodification of food – and food systems – forecloses any recognition of the non-monetised, or *caring*, elements of food (Chapters 2, 3 and 4).^10^

 In their introductory chapter, the four editors define ‘commoning’ as a form of governance that:

“ *differs from the market allocation mechanism based on individual profit maximization and state governance based on command and control. It demands new institutions, goal setting and forms of interaction, thereby forming the bedrock to support a new moral narrative, a new transition pathway, a new economic model and a new relationship with nature and the planet Earth… Commons are not about maximizing individual utilities, selfish individualism or legitimizing the use of force but rather collective decisions, institutions, property and shared goals to maximize everybody’s well-being*” (emphasis added).^10^

There is a strong affinity between this articulation and Dardot’s and Lavel’s theorisation of the politics of the common as ‘instituent praxis,’ as outlined above. Vivero-Pol and his co-editors return to this reasoning in the conclusion, where they argue that the institution of a new governing paradigm – *Food as a Commons* – is not only desirable but essential, due to the manifest failures of both the commodified capitalist food system and the statist bureaucracy that enables it, to fulfil the basic task of feeding humanity on an equitable or sustainable basis.^10^ They go further, to argue that the commons should not be conceived of as merely a third civil society sector co-existing alongside the capitalist market and the state, but rather should be theorised and enacted according to a much more ambitious and transformative political-economic and cultural vision.

How might such a transformative political-economic project be realised? In the first instance, the editors consider the currently existing human right to adequate food, as set forth in the 1966 International Covenant on Economic, Social and Cultural Rights (art.11) and detailed in General Comment 12 of the Committee on Economic, Social and Cultural Rights.^94,95^ Whilst noting that this right, if implemented, would mitigate some of the worst exigencies of the current capitalist food system, it would not question the logic of that system, since the realisation of the right to food is premised on the purchasing of food in commodity market exchanges.^10^ Further, they contend that the individualistic, Western-centric and state-centric nature of human rights discourse and practice can be ‘disempowering’ of community aspirations to advance collective claims for justice.^10^

Contrasting the partial and defensive approach of human rights which ‘lacks the self-instituting dimension of re-commoning,’ the editors cite as examples of emergent *food as commons *practices several social and socio-economic innovations familiar to food system and food movement scholars and practitioners: community supported agriculture, collectively managed community gardens and urban farms, cooperatively managed supermarkets and grocery stores.^10^ Drawing on CB Macpherson’s insight that the ‘possessive individualism’ that patterns and structures contemporary life is at once the cultural, economic and behavioural outcome of centuries of ‘bourgeois society,’^96^ they argue, echoing Federici, that the cultural logic of ‘food as a commons’ is based in ‘[relations of] production and distribution [that] respond to [an ethic] of solidarity and mutual help, rather than to a logic of competition and exclusion.’^10^ Further, and aligned with Dardot and Laval, they contend that these practices and innovations are self-instituting because it is the individuals and communities participating who set the rules.

However, in what appears to be a retreat from the more radical implications of this line of reasoning, the editors conclude with a proposal remarkably similar to Bollier’s concept of the market/state/commons triarchy: the establishment of a ‘tri-centric governance system recombining market rules, public regulations and self-regulated collective actions [which] would combine civic collective actions for food, an enabling state and socially-responsible private enterprises.’^10^ The ‘enabling state’ is posited as a blend of Bollier’s ‘partner state’ and an ‘entrepreneurial state,’ being ‘a shaper and creator of markets and facilitator for civic collective actions to flourish.’^10^ A re-imagined ‘socially-responsible’ for-profit private sector is conceived as a domesticated form of contemporary private business, ‘with a primacy of labour and natural resources over capital’ rather than vice-versa.^10^ The editors expect the transition from market and state hegemony to their ‘real utopia’ of ‘food as commons’ to ‘last for decades;’ they anticipate sustained resistance from ‘Big Food,’ and call for ‘imagination and motivation’ as well as a ‘self-reflective attitude that allows the movement to get stronger and more aware.’^10^

Calls for ‘imagination and motivation’ seem to be a rather tame way to conclude a transformative agenda, and expose, in contrast to Wright, Federici, Dardot and Laval, the lack of a concrete political strategy to ground the visionary ambitions of ‘food as commons.’ This critique is developed by Eric Holt-Gimenez and Ilja van Lammeren in their interrogation of the ‘Food as Commons’ proposal through the framework of food sovereignty.^97^ They note that commons and practices of commoning are historically, culturally and geographically specific, which in turn locates them within wider sets of social relations that frequently reflect asymmetrical distributions of power and access to resources based on gender, class and other distinguishing characteristics.^97^ Further, they note, the commons has had an ambiguous and contradictory relationship with capitalism, being both a ‘means of resistance and a means of exploitation [depending] on the correlation of forces between peasant production and capitalist production.’^97^ Equally, they argue, there is nothing inevitably ‘democratic’ about the commons and its governance: everything depends on the wider social and political context.^97^ Historically, they contend that the commons has not been the ground of revolutionary political action but rather has ‘[provided] a space of resistance for communities attempting to protect themselves from [hegemonic structures of capitalist or liberal-democratic dominance].’ The claims being made by Vivero-Pol and his co-editors rest, according to Holt-Gimenez and van Lammeren, on two ‘co-constitutive assumptions: [first] that food sharing is the material basis for the transformation of the capitalist food regime and [secondly] that the ideational power of discursive democracy is a sufficient driver to refashion the regime’s governance structure.’^97^

These assumptions are problematic for several reasons, all of which point to the essentially idealistic, abstracted and somewhat politically naïve character of the ‘Food as Commons’ proposal. The contention that ‘discursive, deliberative democracy will lead a transformation towards more sustainable and ethical forms of production’ privileges the ‘western eater or food citizen’ as the primary agent of change, which is ‘an uncomfortable projection of consumer politics upon the communities who produce most of the world’s food.’^97^ Similarly, basing transformational change in ‘food sharing [rather] than ownership of the means of production or the redistribution of assets’ also betrays a Western, liberal bias at the heart of the discourse.^97^ Holt-Gimenez and van Lammeren point out that the silence on ‘the roles of land and labour’ in the food as commons tri-centric governance model ‘leaves much of capital’s power intact.’^97^

In contrast to this overly idealised and abstract conception of food as commons, Holt-Gimenez and van Lammeren locate contemporary struggles for the reinstatement or institution of the commons as another ‘chapter [in the] centuries-long, anti-capitalist, class war’ that began with the enclosures of the commons at the dawn of the age of capitalism in the 16th century, and has continued in different forms and places, with varying intensity and outcomes, ever since.^97^ They locate the emergency of food sovereignty – a ‘concept openly grounded in class’ – as similarly an expression of this ongoing struggle.^97^ By way of specific historical example, they cite the anarcho-syndicalism of the peasant-worker militias and cooperatives during the Spanish Civil War (1936-1939), in which:

*“…Tens of thousands of acres of municipal land were worked in common. Money was abolished. Food produced on the collectives was distributed freely and equally through ration cards. Labour, tools, goods and militia members flowed between collectives as needed. Commissions for the production and sale of dairy, livestock, rice, oranges, potatoes and other crops set quotas and arranged distribution…Food commoning was a deeply embedded political strategy constructed within a revolutionary movement for a new, classless society, governed on anarcho-syndicalist principles…Two things become immediately clear: first, that the food as a commons was not the guiding principle but a corollary system of organisation of resources, and secondly, that the practice of food commoning was grounded in the practice of collectivisation, itself part of traditional forms of mutual aid and village-scale Commons regimes.*”^97^

In more recent decades, the land occupation and self-sufficiency praxis of the Brazilian Landless Workers Movement, which has formed over 2000 settlements providing homes and livelihoods to 370000 families, is a prominent exemplar of food de-commodification, commoning and food sovereignty.^97^

This critique is important as it serves as a vital ‘reality check’ for the idealistic claims made for Food as Commons. At the same time, it is important to note that food sovereignty itself is also beset with challenges and contradictions. While it may be ‘openly grounded in class,’ there are nonetheless considerable class tensions and contradictions within the food sovereignty movement, especially between rich and poor peasants, and above all those who are landless.^98,99^ As a farmer-based movement in countries such as Australia, it is characterised by an uncomfortable silence on the question of settler colonialism as well as the privileging of an idealised model of the US-style homestead family farmer that equally appeals to and relies upon wealthy consumers to be sustained (‘vote with your dollar’).^100^

## Conclusion

Until recently, it has for most ‘been easier to imagine the end of the world than to imagine the end of capitalism.’^89^ The COVID-19 pandemic has been a disruptive event, for the food system, for the wider economy, for national and global political elites, and for populations everywhere. Glimpses of a different, quieter, more peaceful and less destructive world have emerged, albeit fleetingly and falteringly. At the same time, the suffering wrought by the pandemic, both directly in the form of disease and death, and indirectly via the cascading economic shocks brought about through society-wide shutdowns, has fallen, and will continue to fall, on the most vulnerable and marginalised members of societies. In many ways it has accelerated and intensified a growing systemic crisis that has been building for decades, politically, economically, ecologically and culturally.

 We have reached a fork in the road. The last time the global capitalist system confronted a systemic crisis was in the 1970s, and that crisis created the conditions for the emergence of neoliberalism, ushering us into the cancer stage of capitalism. The time before that, in the 1930s, the profound economic crisis heralded the rise of genocidal fascism and world war, with tens of millions dead in the worst slaughter humanity has ever unleashed. The embers and echoes of both these earlier decades of systemic crisis are with us now, at the beginning of the 2020s. Capitalism is once more in profound, systemic crisis. The political far right is, once more, in the ascendancy. The drums of war are being beaten, with China the clearly identified ‘enemy.’

 At the same time, the yearning for profound change in the direction of greater equality and ecological integrity is both powerful and substantial, with major political protests in 2019 and 2020 in many parts of the world. Hence the significance, relevance and importance of proposals for transformative change in both food system governance and in the social relations that underpin the food system. Currently we have global and national food systems that are oligopolistic in nature, supported by political structures that resemble plutocracies and oligarchies more closely than they do democracies, insofar as that characterisation is based on their policy development and policy outcomes. Dardot and Laval’s theorisation of the political principle of the common, informed by Holt-Gimenez and van Lammeren’s historically and materially grounded modification of the food as commons proposal, with Federici’s insistence on an explicit anti-capitalist orientation, offers progressive scholars, activists and practitioners a principled and hopeful pathway beyond the contemporary crisis.

## Acknowledgements

 Thank you to Phillip Baker for his support and helpful comments, and thank you to the reviewers for their constructive feedback and comments.

## Ethical issues

 As this article was a literature review based on secondary data, no ethics approval was required.

## Competing interests

 Author declares that he has no competing interests.

## Author’s contribution

 NR is the single author of the paper.
